# The Ambiguous Cue Task: Measurement reliability of an experimental paradigm for the assessment of interpretation bias and associations with mental health

**DOI:** 10.3758/s13428-024-02451-y

**Published:** 2024-07-12

**Authors:** Diana J. N. Armbruster-Genç, Rebecca A. Rammensee, Stefanie M. Jungmann, Philine Drake, Michèle Wessa, Ulrike Basten

**Affiliations:** 1 Department of Psychology, RPTU Kaiserslautern-Landau, Fortstraße 7, 76829 Landau, Germany; 2 Department of Clinical Psychology, Psychotherapy, and Experimental Psychopathology, Johannes Gutenberg-University Mainz, Mainz, Germany; 3 Department of Clinical Psychology and Psychotherapy of Childhood and Adolescence, Johannes Gutenberg-University Mainz, Mainz, Germany; 4 DIPF | Leibniz Institute for Research and Information in Education, Frankfurt am Main, Germany; 5 Department of Clinical Psychology and Neuropsychology, Institute of Psychology, Johannes Gutenberg University Mainz, Mainz, Germany

**Keywords:** Cognitive bias, Psychological well-being, Translational task

## Abstract

Interpretation biases in the processing of ambiguous affective information are assumed to play an important role in the onset and maintenance of emotional disorders. Reports of low reliability for experimental measures of cognitive biases have called into question previous findings on the association of these measures with markers of mental health and demonstrated the need to systematically evaluate measurement reliability for measures of cognitive biases. We evaluated reliability and correlations with self-report measures of mental health for interpretation bias scores derived from the Ambiguous Cue Task (ACT), an experimental paradigm for the assessment of approach-avoidance behavior towards ambiguous affective stimuli. For a non-clinical sample, the measurement of an interpretation bias with the ACT showed high internal consistency (*r*_*SB*_ = .91 – .96, *N* = 354) and acceptable 2-week test–retest correlations (*r*_Pearson_ = .61 – .65, *n* = 109). Correlations between the ACT interpretation bias scores and mental health-related self-report measures of personality and well-being were generally small (*r* ≤ |.11|) and statistically not significant when correcting for multiple comparisons. These findings suggest that in non-clinical populations, individual differences in the interpretation of ambiguous affective information as assessed with the ACT do not show a clear association with self-report markers of mental health. However, in allowing for a highly reliable measurement of interpretation bias, the ACT provides a valuable tool for studies considering potentially small effect sizes in non-clinical populations by studying bigger samples as well as for work on clinical populations, for which potentially greater effects can be expected.

**Supplementary Information:**

The online version contains supplementary material available at 10.3758/s13428-024-02451-y.

## Introduction

Current cognitive models of mental disorders assume that emotional disorders are characterized by cognitive biases in favor of affective information with negative valence (Beck, 1979; Beck & Bredemeier, 2016; Beck & Clark, 1997; Mogg & Bradley, 1998). These biases are considered not only to be a symptom of these disorders but to contribute causally to their onset and maintenance (Everaert et al., 2015; Rude et al., 2003; Beck & Clark, 1988; Mathews & Mackintosh, 2000), constituting a cognitive vulnerability to emotional disorders (Mathews & MacLeod, 2005). Over decades, a large number of empirical studies have investigated such biases in information processing and linked them to clinical diagnoses, psychopathological symptoms, or personality traits of positive-versus-negative effect (for reviews see Cisler et al., 2009; Everaert et al., 2017). While many studies have reported findings supporting the assumption of cognitive biases, particularly in anxiety disorders / high trait anxiety and depressive disorders / high trait depressiveness, there have also been null findings reported time and again (for a review, e.g., see Cisler et al., 2009).

Over the last years, it has become apparent that many studies in the field have used experimental paradigms that do not allow for a reliable measurement of individual differences in cognitive biases in the first place (Rodebaugh et al., 2016; Hedge et al., 2018). As a result, a broad range of previous reports that did not take into account the reliability of bias assessment were called into question (McNally, 2019). The field has since recognized that more attention needs to be paid to the reliability of bias measurements in experimental psychopathology and clinical research (MacLeod et al., 2019; Parsons et al., 2019a). While for paradigms used to measure individual differences in attention biases, a number of studies have already reported estimates of measurement reliability (e.g., Schmukle, 2005; Kappenman et al., 2014; Skinner et al., 2018) and reliable alternatives to traditional measures have been developed (e.g., Lazarov et al., 2018), for measures of interpretation biases, the question of measurement reliability is still largely open.

For this study, we focused on an experimental paradigm designed to measure individual biases in the interpretation of ambiguous information that stems from animal research and has been adapted for use in humans: the Ambiguous Cue Task (ACT; Harding et al., 2004; Mendl et al., 2009; Schick et al., 2013). We used the ACT to measure individual interpretation biases in a large sample of adult human subjects (*N* = 353). As estimates of reliability, we report indices of internal consistency and retest correlations. To address the question of measurement validity, we further examine how the interpretation bias measure from the ACT relates to self-report measures of personality and well-being associated with mental health. Our study is intended to assess the suitability of the ACT for the assessment of individual differences in interpretation biases in experimental psychopathology and clinical research. Ensuring measurement reliability is an important prerequisite to use the task for further – more reliable – research on the relevance of interpretation biases for the development and maintenance of affective disorders.

Individual biases in interpreting ambiguous affective information have been an important research focus with regards to anxiety and depressive disorders (Everaert et al., 2017; Hindash & Amir, 2012). Complementing psychopathology-focused research, recent accounts of psychological resilience have proposed processes of stimulus interpretation (especially positive appraisal and reappraisal processes) to play an important role for an individual’s capacity to maintain mental health in the face of severe adversity (Kalisch et al., 2015). Previous research has used many different approaches to assess individual interpretation biases (see Schoth & Liossi, 2017 for an extensive review). All approaches have in common that study participants are presented with affectively ambiguous stimulus material. Dependent measures are collected reflecting the individual interpretation of the ambiguous material as rather positive or negative, with some variety in the exact indices recorded (e.g., individually formulated interpretations, choices from preformulated interpretations, reaction times for the formulation or choice of interpretations, or choices/response times reflecting approach vs. avoidance behavior). Studies also vary in the specific types of ambiguous stimuli used (e.g., words, images, scenarios, or sounds).

The variety of existing measures can be divided into *direct* and *indirect* measures of interpretation bias (De Houwer, 2006; De Houwer & Moors, 2010). For a *direct* measure, participants are asked to self-assess the attribute that is supposed to be measured. In the case of an interpretation bias, this applies to measures that result from asking participants to directly rate their own tendency for negative (or positive) interpretations. For an *indirect* measure, on the other hand, participants produce responses, which are interpreted by researchers to infer the underlying attributes that are assumed to influence the responses. Indirect measures of interpretation biases typically measure approach-avoidance decisions for ambiguous stimuli that are assumed to be influenced by stimulus interpretations. Direct measures have been criticized for being prone to demand effects, as it is easy for participants to infer the measure of interest. Consequently, social desirability or assumptions about potential study objectives could affect the participants’ responses. In contrast, indirect measures are usually less transparent and therefore less susceptible to strategic influences. In addition, they offer potential for translational use in human and animal research. Another classification distinguishes between *online* and *offline* measures of interpretation bias (Mathews & MacLeod, 2005). While *online* measures record interpretations made right at the time a person encounters ambiguous information, *offline* measures represent judgements about interpretations that people have made in the past, expect for the future, or hypothetically assume for themselves. The ACT allows for an *indirect online* assessment of interpretation biases.

The Ambiguous Cue Task (ACT) was first presented as an experimental paradigm to assess affective states in rodents (Harding et al., 2004). The paradigm has subsequently been used for the study of cognitive biases in different species (for a review see Mendl et al., 2009) and also been adopted for the study of interpretation biases in humans (e.g., Schick et al., 2013, 2015; Paul et al., 2011; Mayer et al., 2022; Iigaya et al., 2016; Wessa, Armbruster-Genç et al., 2023). The common principle of the ACT is that in a first step, during an acquisition (or conditioning) phase, previously neutral stimuli become associated with positive and negative outcomes. Participants learn to approach positively associated stimuli with certain perceptual characteristics (e.g., a high tone) and to avoid negatively associated stimuli with different perceptual characteristics (e.g., a low tone). In a second step, during a test phase, participants are then presented with both the (unambiguous) positive and negative stimuli from the acquisition phase as well as with stimuli that vary in their degree of ambiguity – including a perfectly ambiguous stimulus which represents the exact middle ground between the positive and the negative stimulus in terms of sensory perception. The measure of interest is the frequency of approach decisions across a number of trials, as signaled by lever or button presses, shown for the ambiguous stimuli. Interpretation biases are thus inferred from evaluating approach vs. avoidance decisions regarding ambiguous stimuli presented in the test phase of the ACT paradigm.

In animal research, the paradigm has been used to study animal well-being. It has repeatedly been shown that animals whose well-being was presumably impaired because they were kept under deprived or unpredictable housing conditions showed indicators of more negative interpretations of the ambiguous stimuli, i.e., fewer approach responses for ambiguous stimuli and longer response time for approach responses, in comparison to animals kept under standard conditions (Harding et al., 2004; Mendl et al., 2009). The studies that have adapted the ACT paradigm for use in humans have shown slight variation in the specific task designs using either auditory (e.g., Anderson et al., 2012; Schick et al., 2013, 2015) or visual stimuli (e.g., Iigaya et al., 2016; Mayer et al., 2022). Paralleling the findings from animal studies, also studies with humans showed that – across participants – interpretation biases varied with experimental conditions: Manipulations designed to negatively affect the participants' mood resulted in more negative interpretation biases than control conditions not manipulating mood (Iigaya et al., 2016; Lin et al., 2019).

Individual interpretation bias scores from the ACT have been reported to correlate with self-reported personality traits and markers of mental health. For instance, Schick et al. (2013) reported more positive interpretation biases to be associated with less reflective pondering, a type of rumination (*r* = – .50, *N* = 20). This association could not be replicated in a second study which, however, reported an association with trait anxiety (*r* = + .44, *N* = 24; Schick et al., 2015). Further studies on personality-related differences in the ACT interpretation bias suggest that a more negative bias (and a less positive bias, respectively) is associated with higher levels of trait anxiety (*r* = +.33, *N* = 38; Anderson et al., 2012) as well as with higher levels of trait negative and lower levels of trait positive affect (*r* = +.43 and *r* = – .33 respectively, *N* = 67; Paul et al., 2011). However, in a more recent study in a larger non-clinical sample (*N* = 113), Wessa et al. (2023a, 2023b) did not find any associations between the ACT interpretation bias and mental health-related measures from self-report, including depressiveness, anxiety, and well-being (all *r* ≤ |.11|). A group comparison of adults with trait dysphoria with a non-dysphoric control group showed significantly higher levels of negative interpretations in the dysphoric group (Lin et al., 2019). However, comparing adolescent ADHD patients with comorbid depression to a group of healthy control subjects, Mayer and colleagues (2022) did not find a significant difference in interpretation bias scores – nor significant correlations between ACT interpretation bias scores and the level of ADHD or depression symptoms. Overall, regarding individual differences in interpretation biases and their association with measures of personality and mental health, the available empirical evidence is partly inconsistent. This may be due to the fact that many of the previous studies investigated rather small samples and therefore had low statistical power to detect small to moderate correlations (the correlational findings summarized above result from studies including between 20 and 140 participants). However, the inconsistent findings across studies could also be explained by a possible lack of reliability in the measurement of interindividual differences in interpretation bias.

Measurement reliability has important implications for a measure’s validity and interpretability. If reliability is unknown, it is near impossible to discern true effects from measurement error, which in turn can lead to inconsistent results across studies and issues with replicability (Parsons et al., 2019a). A prominent example from the bias literature is the Dot Probe Task, which has been used in numerous studies to investigate attentional biases in the processing of affective information in both clinical and non-clinical samples (e.g., MacLeod et al., 1986; Broadbent & Broadbent, 1988; Mansell et al., 1999; Bradley et al., 1999; Bar-Haim et al., 2007). The mixed results obtained with this task (for a review see e.g., Cisler et al., 2009) could be attributed to insufficient reliability of measurements with the Dot Probe Task (Schmukle, 2005; Staugaard, 2009; MacLeod et al., 2019). This example demonstrates how important it is to assess and report reliability estimates for experimental measurements of individual differences in cognitive biases. These must be taken into account when interpreting correlations with measures of personality or mental health.

If the ACT is to be used in experimental psychopathology and clinical research for correlative studies on the association between interpretation bias and personality/mental health, then two conditions must be met: (a) the task must allow reliable measurement of individual levels of interpretation bias, and (b) the samples used to study associations with personality / mental health must be large enough to allow detection of effect sizes of interest with sufficient statistical power. Here, we report findings for a non-clinical sample of mainly university students (*N* = 354), large enough to detect correlations ≥ .15 with 80% power. For our measurement of interpretation bias with the ACT, we report reliability estimates of internal consistency (Cronbach’s alpha and permutation-based split-half) and two-week test–retest correlations [Pearson, ICC (2,1), and ICC (3,1)] . Furthermore, to assess the validity of the ACT interpretation bias scores, we calculated correlations with mental health-related self-report measures of personality and well-being. We expected more positive scores of an ACT interpretation bias to be associated with higher levels of trait positive affect, optimism, well-being, and resilience and lower levels of trait negative affect, pessimism, anxiety, and depressiveness. Considering that effects in non-clinical populations might only be evident in individuals showing extreme expressions of the variables of interest, we also conducted analyses for extreme groups regarding our measures of bias.

## Methods

### Sample size justification

In the sample size planning, we focused on the analysis that required the largest sample. This was the correlational analysis of the association between ACT interpretation bias scores and the mental health-related self-report measures of personality and well-being, used to evaluate association with mental-health related measures. For this, sample size calculations were based on a *smallest effect size of interest* (Lakens, 2022; Lakens et al., 2018). For the specific research question at hand, there is little previous research that would suggest effect sizes to be expected, and the few previous findings that have been reported (see Introduction) are probably not suitable for deriving reliable estimates of effect sizes due to the small samples in which they were obtained. From other studies in other domains of psychology, we know that correlations between *implicit* behavioral measures (as acquired in the ACT) and explicit self-report measures (as assessed with questionnaires) are typically rather small, rarely exceed effect sizes of *r* = .25, and are often considerably lower (Mischel, 1968; Bernoster et al., 2019; Bosco et al., 2015). We therefore wanted to ensure that we could detect correlations well below .25 with decent statistical power. On the other hand, very small correlations close to zero did not appear to be of theoretical interest to us. Taking these two aspects into account, we defined .15 as the *smallest effect size of interest* for this study. For this *smallest effect size of interest*, sample size calculations suggested a sample size of *N* = 345 to be necessary to detect correlations of *r* ≥ .15 with 80% power at a *p* level of .05 in two-sided tests (R package *pwr*; Champely, 2020).

Separate sample-size calculations were conducted for the reliability analyses. Here, we expect much larger correlations. Reliability estimates should be greater than *r* = .60 for the measurement of question to be anywhere near satisfactory. One might want to be able to derive reliable estimates even for coefficients falling below this threshold to be able to evaluate measurement reliability if it falls in the range of .40 to .50. However, correlations smaller than .40 would be of little practical interest anymore because such low measurement reliability would generally be considered clearly insufficient. For the reliability analyses, the *smallest effect size of interest* was therefore set to .40. Here, sample size calculations suggested a sample size of *N* = 46 to be necessary to detect correlations of *r* ≥ .40 with 80% power at a *p* level of .05 in two-sided tests. Thus, for the reliability analyses, a significantly smaller sample was sufficient than for the correlational analyses conducted to evaluate associations with mental health related measures. We took this difference into account in data acquisition.

### Participants

For the correlational analyses relating ACT bias scores and self-report measures, taking into account a 5–10% dropout, we collected data from a total of 378 participants. Data were collected in three waves (*n* = 123, *n* = 180, *n* = 75) between January 2019 and July 2021. Regarding the experimental data from the ACT, 22 participants were excluded from analyses due to missing data in more than 20% of the trials or less than 70% correct answers in the positive and negative reference trials of the ACT (signaling either poor motivation or poor understanding of the task). Another two participants were excluded from the analyses due to prior knowledge of the task and technical problems during data recording. The final sample of *N* = 354, for which ACT data was available, included 223 female, 118 male, two diverse participants, and 11 participants that did not give gender information. Age ranged from 18 to 60 years (*M* = 23.7, *SD* = 5.4). Questionnaire data were available for 352 of these 354 participants. Consequently, analyses considering only the experimental data from the ACT were based on data from *N* = 354 participants, while correlational analyses relating the experimental data from the ACT to the questionnaire data were based on data from *N* = 352 participants.

For the reliability analyses, we also collected retest data for the ACT after a 14-day test–retest interval (+/– 1.2 days), but only in the first wave of data collection, resulting in retest data being available for a subsample of *n* = 109 participants, after exclusion of cases according to the criteria described above. This subsample included 60 female, 43 male, and six participants without gender information. Age ranged from 18 to 60 years (*M* = 24.0, *SD* = 6.6). The study was approved by the local ethics committee of the University of Frankfurt (ID 2016-20). Recruitment routes included advertisements via flyers, emails, social media, and personal contacts and were primarily addressed to university students. Participants were compensated for participation with course credit or monetary compensation.

### Paradigm

Individual biases in the interpretation of ambiguous affective information were assessed with an Ambiguous Cue Task (ACT). The task version used in this study is an adaptation of a paradigm from animal research (Harding et al., 2004). We used the task structure established in previous studies with humans. The task was programmed in Presentation® (www.neurobs.com) and conducted in person in the lab. It comprised two parts: (a) an *acquisition* phase, in which participants learned the task with feedback and (b) a *test* phase, in which participants performed the task without feedback (task code available on OSF: https://osf.io/h5p89/?view_only=16d56cdd59424f0ea429bbb252801303). The task was presented on an LCD screen of 47.2 × 29.5 cm size with a resolution of 1680 × 1050 pixels. Participants were placed at an approximate distance of 60 cm from the screen, ensured by the monitor and the participant’s chair having fixed positions marked on desk and floor and participants being asked to sit upright and not lean forward or backward. Participants were instructed that in each of the two runs of the test phase, they could win a maximum of 12 euros and that they would be paid out their gains for the run with the better result. Detailed task instructions can be found in the SI.

**Fig. 1:**
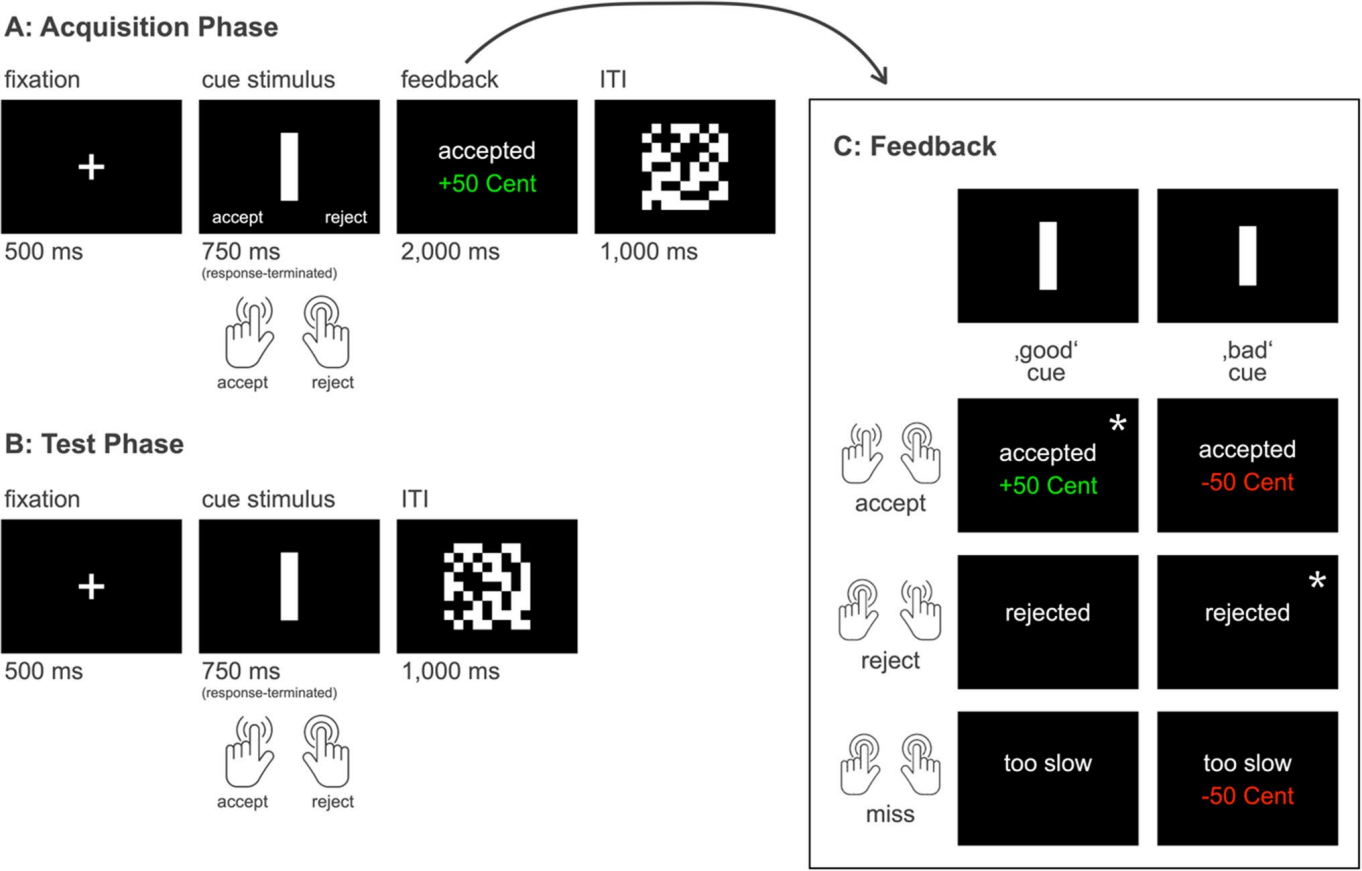
Illustration of the Ambiguous Cue Task (ACT). Schematic illustration of the trial structure **A:** in the acquisition phase and **B:** the test phase (without feedback). **C:** Feedback matrix used in the acquisition phase. Asterisks (*) highlight the correct responses for the 90% accuracy criterion

*Acquisition phase.* In the acquisition phase, participants learned to associate abstract visual stimuli (white bars on a black background) with monetary consequences. Longer bars (length: 5.34, 5.5, 5.67 cm) bars were associated with positive consequences, i.e., a monetary gain of + 50 cents, and shorter bars (length: 4.37, 4.5, 4.64 cm) with negative consequences, i.e., a monetary loss of – 50 cents (or vice versa, counterbalanced across subjects). All bar stimuli had a width of 1 cm. Figure 1A illustrates the trial structure in the acquisition phase. At the beginning of each trial, a fixation cross was presented for a duration of 500 ms. Following the fixation cross, the cue stimulus was presented for a maximum duration of 750 ms. The participants had to respond to the stimulus within this 750-ms response window by pressing either of two keys on a standard computer keyboard using their left and right index fingers, “C” to accept or “M” to reject (counterbalanced across participants). The participant’s response terminated the presentation of the target stimulus. After offset of the cue stimulus, feedback was presented for 1500 ms. If the participant had responded within the presentation interval of the target stimulus, the feedback reflected the participant’s choice as “accepted” or “rejected” (a miss was followed by the feedback ‘too slow’). In case the participant had accepted the stimulus, the feedback further informed the participant about the monetary consequences of the decision, i.e., if the stimulus was associated with a 50 cents gain (‘+ 50 cents’ in green font, RGB [29, 177, 10]) or a 50 cent loss (‘– 50 cents’ in red font, RGB [221, 0, 2]; see Fig. 1B for a schematic illustration of the feedback matrix).

After the feedback, a mask stimulus was presented for 1000 ms. The mask was introduced to prevent spill-over of stimulus presentations from one trial to the next in the main task, where (without the feedback screen) cue stimuli would otherwise only be separated by the 500 ms presentation of the fixation cross. In random order, 20 different mask stimuli, generated as QR code, were presented with a size of 11 × 11 cm^2^. The assignment of long versus short bars to ‘good’ versus ‘bad’ consequences was counterbalanced across participants, as was the assignment of left and right response buttons to ‘accept’ versus ‘reject’ decisions.

In the acquisition phase, every participant completed a minimum of two blocks with 30 trials each, in which the six different stimuli used in the acquisition phase (three long and three short bars) were presented five times each. Training stopped after two blocks if participants had reached the criterion of ≥ 90% correct answers (rejection of ‘bad’ bars and acceptance of ‘good’ bars) in the second block of the training. If performance was < 90% accuracy, participants were presented with up to three more blocks of the task until they reached the criterion. At the end of each block, the participants received feedback on the sum of money they had collected. These amounts were not paid out.

*Test phase.* In the test phase participants performed two runs of the task without feedback. Now they were presented with stimuli of five different length. Two lengths that the participants were already familiar with from the acquisition phase, a long bar (5.5 cm) and a short bar (4.5 cm), served as positive reference (PR) and negative reference (NR) stimuli. In addition, in the test phase, participants were presented with stimuli of intermediate length: A fully ambiguous (AM) cue, which was 10% shorter than the long reference bar and 10% longer than the short reference bar (4.95 cm), and two partly ambiguous stimuli that were 7% shorter than the long bar (5.12 cm; near positive, NP) or 7% longer than the short bar (4.82 cm; near negative, NN).

Each run of the task consisted of four blocks, which comprised 30 stimuli (each of the five bar stimuli used in the test phase was presented six times in each block). Stimulus presentation times and sequence were the same as in the acquisition phase. However, the trial-wise feedback was omitted. The first 20 trials of the test phase were still regarded as part of a training to allow for accommodation to the fact that there was no feedback anymore. These first 20 trials were excluded from the analysis. Consequently, all analyses reported in the following were based on data from 220 trials.

### Self-report questionnaires

Participants completed a range of self-report questionnaires assessing individual differences in mental-health related personality traits and well-being via the online platform SoSci Survey (http://www.soscisurvey.de). The questionnaires included the Resilience Scale (RS-25; Wagnild & Young, 1993), the Life Orientation Test – Revised (LOT-R; Scheier et al., 1994), the Behavioural Inhibition System and Behavioural Activation System Scale (BIS/BAS; Carver & White, 1994), the Positive and Negative Affect Schedule (PANAS; Watson et al., 1988), the State-Trait Anxiety-Depression Inventory (STADI; Laux et al., 2013), and the World Health Organization Well-Being Index (WHO-5; World Health Organization, 1998). Two of these questionnaires (RS-25, WHO-5) consist of just one total scale, the other four (PANAS, STADI, LOT-R, BIS/BAS) each comprise two sub scales. The resulting ten scale scores can be grouped into those that reflect positive affectivity and are linked to better mental health (Mental Health +) and those that reflect negative affectivity and are linked to poorer mental health (Mental Health –). The Mental Health + factors, conceptually linked to better mental health, include: trait optimism (LOT-R), trait resilience (RS-25), well-being (WHO-5), trait positive affect (PANAS), and behavioural activation (BIS/BAS). The Mental Health – factors, conceptually linked to poorer mental health, include: trait pessimism (LOT-R), depressiveness and anxiety (STADI), trait negative affect (PANAS), and behavioral inhibition (BIS/BAS).

### Procedure

Data for the ACT was collected within three bigger studies on cognitive bias and mental health, together with other experimental and questionnaire data that will be reported elsewhere. The ACT was administered by trained research staff in the lab. The online questionnaires on personality and well-being were completed from home after participants had completed the lab-based data collection for the ACT. This study was not preregistered.

### Analyses

All analyses were performed in RStudio, Version 2022.7.1. Data and analysis scripts can be found at https://osf.io/h5p89/?view_only=ee139c1afe754633bc29d7923f90e6a4.

*ACT: Choice Behavior Across Participants.* To investigate choice behavior across participants, we analyzed acceptance rates, ranging between – 1 (100% rejected decisions) and + 1 (100% accept decisions) as depending on stimulus type (PR, NP, AM, NN, NR) in a one-way ANOVA.

*ACT: Calculation of Individual Interpretation Bias Scores.* There are two possible ways to assess individual interpretation bias with the ACT: (i) calculated as the mean of all accept responses (coded as 1) and all reject responses (coded as – 1) using only *fully ambiguous* stimuli (in the following referred to as the standard score for ‘interpretation bias – core’ or ACT-IB-core) or (ii) calculated as the mean of all accept responses (coded as 1) and all reject responses (coded as – 1) using *fully ambiguous* stimuli as well as *partly ambiguous* stimuli (near positive and near negative) (in the following referred to as ‘interpretation bias – extended’ or ACT-IB-extended). Responses to fully ambiguous trials are conceptually more clearly related to potentially pre-existing biases of interpretation that come into play when perceptual features of a stimulus give no hint as to whether the stimulus is more likely to be associated with positive or negative consequences. The partly ambiguous stimuli (NP and NN), on the other hand, still contain some discriminatory information making them more likely to be associated with positive vs. negative consequences. Nevertheless, they can incite biased interpretations. To provide a basis for the decision on which score to use, we report psychometric properties for both the *interpretation bias *–* core* and *the interpretation bias – extended*.

*Reliability of the ACT Interpretation Bias. T*o evaluate the internal consistency of the individual interpretation bias measure from the ACT, we calculated Spearman–Brown-corrected mean split-half correlations for 10,000 permutation-based splits of the experimental data (R package: splithalf; Parsons, 2021). In addition, for comparability with other studies, we report Cronbach’s alpha (R package: psych; Revelle, 2022). To evaluate test–retest correlations, we used data from a subsample with ACT data from two measurement occasions that were 2 weeks apart (n = 109). Here we report (a) the Pearson correlation coefficient between scores from T1 and T2 and (b) intraclass correlation coefficients (ICC; Shrout & Fleiss (1979), both calculated using the *psych* package (Revelle, 2022). While the ICC(2,1) quantifies *absolute agreement*, the ICC(3,1) reflects *consistency* across measurement occasions.

*Validity of the ACT Interpretation Bias.* To investigate associations of interpretation biases with the mental health-related self-report measures of personality and well-being, Pearson correlations were calculated between the individual ACT interpretation bias scores and all ten scale scores from the self-report questionnaires, of which five are conceptually linked to better mental health (Mental Health + group) and five to poorer mental health (Mental Health – group), see above.

*Extreme groups.* To further explore the relation between the ACT interpretation bias and the mental health-related self-report measures, we examined extreme groups in our large non-clinical sample (*N* = 354) based on the ACT interpretation bias scores. For this, we selected the upper and lower 10% of participants, i.e., participants with the most positive and the most negative interpretation bias scores. The proportions of 10% each were chosen as a compromise in order to both ensure extreme bias scores and reasonable group sizes. For the ACT-IB-core, this resulted in a comparison of *n* = 36 participants showing extreme positive biases with *n* = 35 showing extreme negative biases. For the ACT-IB-extended, the group sizes were *n* = 35 and *n* = 36, respectively. Post hoc calculations suggest that in two-sided tests, groups of *n* = 35 allow to detect differences of *d* ≥ 0.68 at *p* = .05 with 80% power.

## Results

### Ambiguity and choice behavior in the Ambiguous Cue Task

**Fig. 2:**
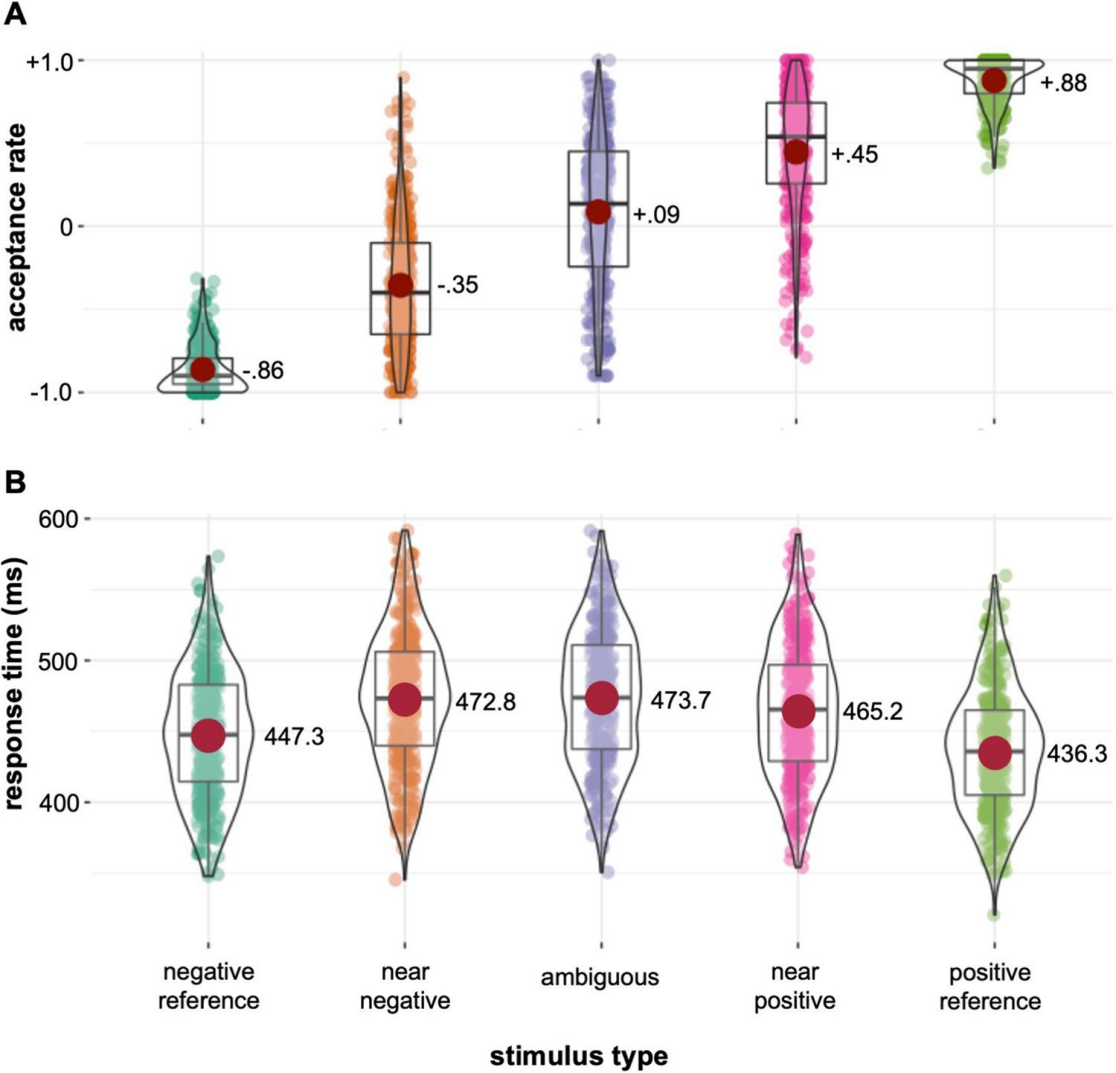
Choice behaviour in the Ambiguous Cue Task. **A** Acceptance rates and **B** response times by stimulus type*. *Red dots and numbers show mean values across subjects

Across participants, we observed a highly significant effect of stimulus type on acceptance rate in the ACT (one-way ANOVA *F*(4,1765) = 161.9, *p* < .001, *η*^*2*^ = .78; see Fig. 2A): The average acceptance rate was – .86 (*SD* = 0.14) for the negative reference stimulus and +.88 (*SD* = 0.14) for the positive reference stimulus. For the partly ambiguous stimuli, we observed intermediate acceptance rates of – .35 (*SD* = 0.40) for the near negative and + .45 (*SD* = 0.39) for the near positive. The mean acceptance rate for the fully ambiguous stimulus was + .09, showing a wide variation across participants ranging from – .90 to + 1.00 (*SD* = 0.46). The averaged mean acceptance rate across all three ambiguous stimuli was +.06 (SD = 0.39). Acceptance rates for the ambiguous stimuli will be referred to as interpretation biases in the following. The mean interpretation bias for the fully ambiguous stimuli was significantly different from 0 (*t*(352) = 3.53, *p* < .001, *d* = – .19). Also response times in the ACT were significantly affected by stimulus type (*F*(4,1765) = 49.9, *p* < .001), showing a slowing of response times with increasing ambiguity of stimuli (see Fig. 2B).

### Reliability of interpretation bias measurement

**Fig. 3:**
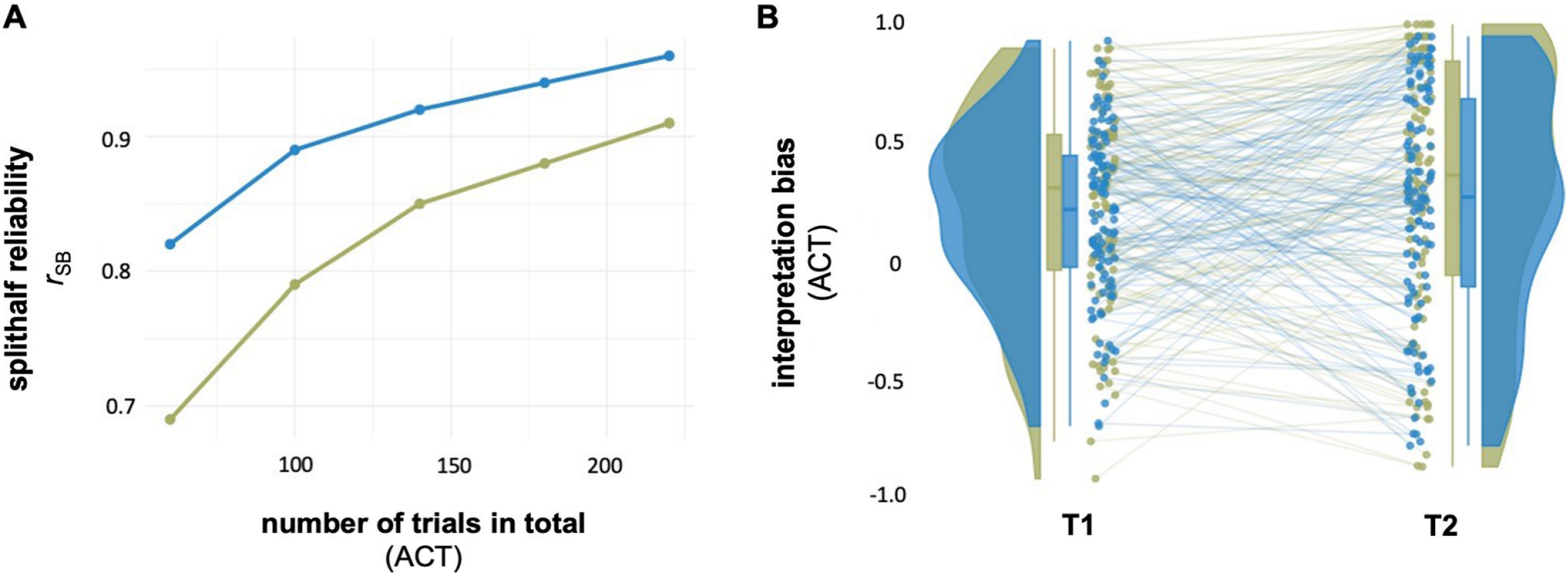
Measurement **r**eliability for ACT **interpretation bias scores.** A Spearman**–**Brown-corrected mean split-half correlations (r_SB_) as depending on ACT trial numbers. B Test (T1) and retest (T2) scores (*n* = 109). *Green*: Interpretation bias – core (ACT-IB-core) based on only fully ambiguous stimuli (AM). *Blue*: Interpretation bias – extended (ACT-IB-extended) based on fully and partly ambiguous stimuli (AM, NP, NN). See SI for separate figures for ACT-IB-core and ACT-IB-extended (Fig. S3)

Table 1 shows the results of the reliability analyses for both ACT interpretation bias scores – core and extended. Estimates for Cronbach’s alpha as well as the Spearman–Brown-corrected permutation-based mean split-half correlation ranged between .91 and .96 and indicated a high level of internal consistency (see also Fig. 3B) and increased with trial numbers included in the calculation of the scores (illustrated for the split-half correlations in Fig. 3A, SI Table S2). As for the same number of total task trials, the ACT-IB-extended score considers more trials than the ACT-IB-core, estimates for the former are generally higher. The test (T1) and retest (2) scores for the ACT interpretation biases are illustrated in Fig. 3B (subsample *n* = 109). Test–retest correlations ranged between .61 and .69 (see Table 1).

**Table 1:** Reliability estimates for the interpretation bias scores (core and extended) from the Ambiguous Cue Task

	ACT-IB-core		ACT-IB–extended	
Reliability	point estim.	[95% CI]	point estim.	[95% CI]
**Estimates of internal consistency** (*N* = 354)				
Cronbach’s alpha	.91	[.90; .93]	.96	[.95; .96]
Mean split-half correlation (Spearman Brown-corr.)	.91	[.89; .92]	.96	[.95; .96]
**2-week test–retest correlations** (*n* = 109)				
Pearson’s *r*	.63	[.51; .73]	.69	[.58; .78]
ICC(2,1) absolute agreement	.61	[.47; .71]	.66	[.54; .75]
ICC(3,1) consistency	.61	[.48; .72]	.66	[.54; .75]

## Correlations with mental health-related self-report measures

**Fig. 4:**
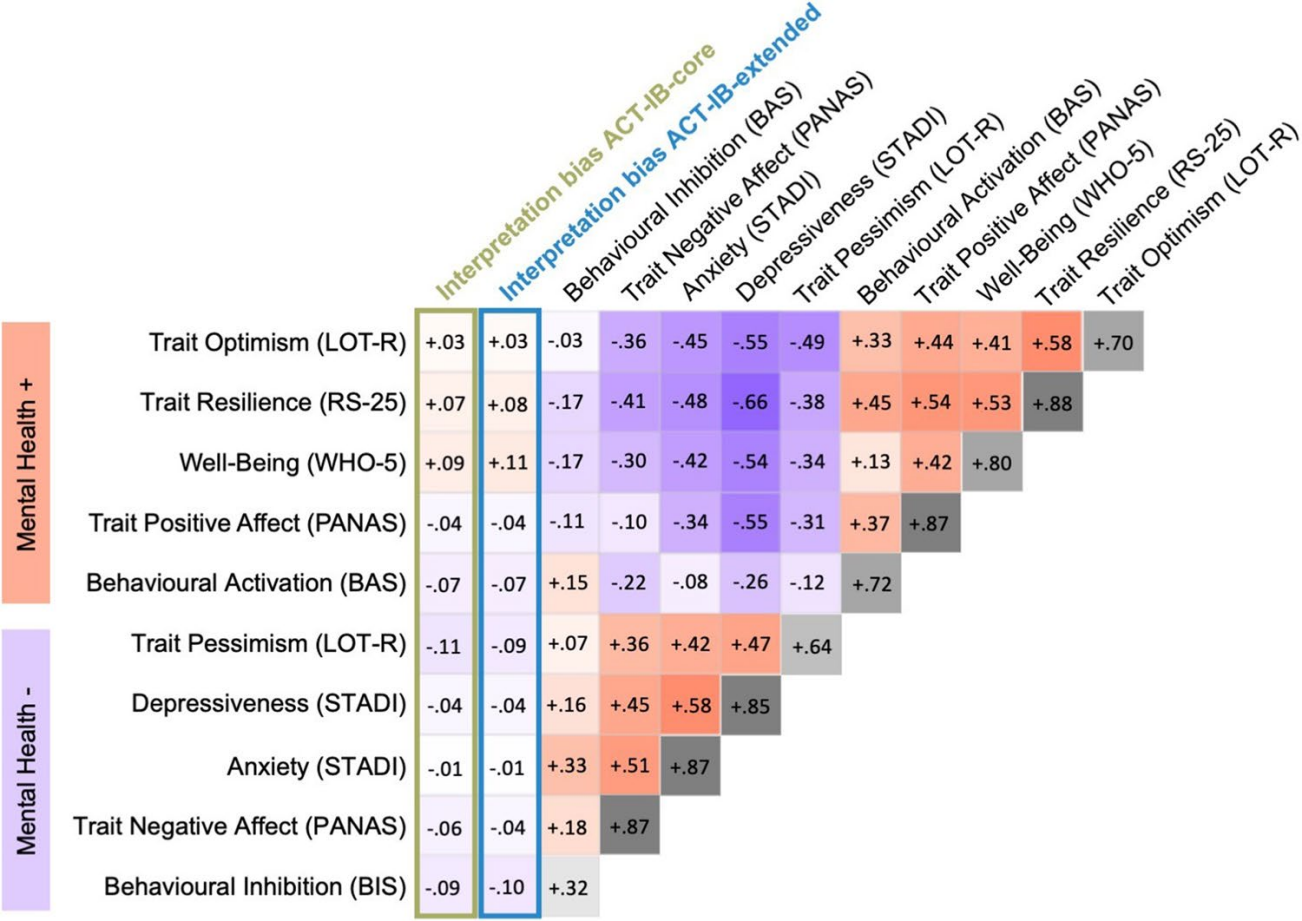
Pearson correlation coefficients between ACT interpretation bias scores and self-report measures of mental health. To facilitate the overview, self-report measures are grouped into those that are conceptually linked to mental health in a positive (Mental Health +) vs. negative (Mental Health -) way. Red and blue indicate positive and negative correlations, respectively. The diagonal shows Cronbach’s alpha for the self-report scores. For *p* values and confidence intervals, see SI Table S2. Correlations involving bias scores were statistically not significant when applying a Bonferroni correction for multiple comparisons (corrected *p* value for ten tests: .005)

To evaluate the validity of the ACT score as a measure of interpretation bias, we calculated correlations with mental health-related self-report measures of personality and well-being (Fig. 4). Overall, although most associations showed the expected directions, effect sizes were small and correlations did not exceed the threshold for statistical significance. Relatively highest coefficients were observed for a positive correlation between the ACT interpretation bias – extended and well-being as assessed with the WHO-5 scale (*r* = .11) and a negative correlation between the ACT interpretation bias – *core* and trait pessimism as assessed with the LOT-R pessimism scale (*r* = – .11). The significance tests for these correlations did, however, not survive a Bonferroni correction for multiple comparisons (corrected *p* value for ten tests: .005).

## Extreme group analyses

In addition to the correlations analyses, within our large sample, we analyzed extreme groups (upper and lower 10%) regarding the interpretation bias score ACT-IB-core. Comparing participants with the most positive interpretation biases (*n* = 36; mean ACT-IB-core = +0.78) to those with the most negative interpretation biases (*n* = 35; mean ACT-IB-core = – 0.75), we observed a tendency for higher well-being (effect size Cohen’s *d* = + .48) and lower depressiveness (*d* = – .49) that did, however, not manifest as significant group differences when applying a Bonferroni correction for multiple comparisons (corrected *p* value for ten tests: .005; see Table 2). Groups did not significantly differ in age (*t*(64) = 0.32, *p* = .751) or sex (*Χ*^*2*^(1) < 0.1, *p* = .877). A grouping by extremes of the interpretation bias score ACT-IB-extended also showed no significant differences in the self-report measures of mental health (details see SI, Table S3).

**Table 2:** Comparison of mental health-related self-report measures for groups with extreme positive vs negative interpretation biases (ACT-IB-Core)

	Extreme negative ACT-IB-core *n* = 34	Extreme positive ACT-IB-core *n* = 32	Group comparison			
	Mean (SD)	Mean (SD)	*t*(*df*)	*p*	95% CI	d
**Mental Health +**						
Trait Optimism (LOT-R)	8.51 (2.22)	8.47 (2.22)	.08 (69)	.937	[– 1.01; 1.09]	-.02
Trait Resilience (RS-25)	129.23 (17.54)	134.00 (18.48)	– 1.12 (69)	.269	[– 13.31; 3.76]	.27
Well-Being (WHO-5)	12.77 (4.06)	14.64 (3.79)	– 2.00 (69)	.049	[– 3.73; – .009]	.48
Trait Positive Affect (PANAS)	35.17 (6.75)	36.36 (6.26)	– .77 (69)	.444	[– 4.27; 1.89]	.19
Behavioural Activation (BAS)	3.26 (.41)	3.10 (.32)	1.83 (69)	.072	[– .014; .33]	-.44
**Mental Health –**						
Trait Pessimism (LOT-R)	4.51 (2.59)	3.64 (1.64)	1.70 (69)	.093	[– .15; 1.90]	-.41
Depressiveness (STADI)	19.29 (4.08)	17.25 (4.30)	2.02 (69)	.045	[.05; 4.02]	-.49
Anxiety (STADI)	21.43 (6.45)	20.42 (4.95)	0.74 (69)	.460	[– 1.71; 3.73]	-.18
Trait Negative Affect (PANAS)	18.14 (6.54)	18.00 (7.12)	.09 (69)	.930	[– 3.09; 3.38]	-.02
Behavioral Inhibition (BIS)	2.94 (.44)	2.81 (.39)	1.39 (69)	.170	[– .06; .33]	-.33

All differences between groups were statistically not significant when applying a Bonferroni correction for multiple comparisons (corrected *p* value for ten tests: .005).

## Discussion

The current study shows that the Ambiguous Cue Task (ACT) is a reliable measure of interpretation bias, both in terms of internal consistency as well as test–retest correlations. In our non-clinical sample, the ACT interpretation bias showed only weak associations with mental health-related self-report measures of personality and well-being (effect sizes *r* ≤ |.11|) that were statistically not significant when controlling for multiple comparisons. Correspondingly, extreme group analyses also showed only small differences between subgroups of participants with particularly strong positive vs. negative interpretation biases (effect size *d* ≤ |.49|) that were also not significant with correction for multiple comparisons. These findings indicate that in non-clinical populations individual interpretations of ambiguous information measured with the ACT do not show clear associations with mental health.

## The Ambiguous Cue Task enables a reliable measurement of individual differences in interpretation bias

In this study, the ACT provided a reliable measurement of individual differences in choice behavior facing ambiguous stimuli that can be interpreted as reflecting differences in interpretation biases. For the full version of the ACT with 240 trials (execution time: 15–20 min plus 15 min for instruction and training) the estimates of internal consistency were all greater than .90, indicating excellent internal consistency. This still applies when trial numbers (and thus execution time) are significantly reduced. With 140 task trials in total, the ACT-IB-core, which only considers decisions for fully ambiguous stimuli (AM), still achieved an *r*_*SB*_ of .85. For an even greater reduction in the number of trials, we recommend using the ACT-IB-extended, which takes into account three different types of trials, including fully ambiguous (AM) and partly ambiguous stimuli (NP, NN). For this score, 60 task trials in total (execution time: 5 min) were sufficient to still achieve an *r*_*SB*_ of .82.

The test–retest correlations were all above .60. The fact that the estimates for ICC(2,1) and ICC(3,1) did not differ suggests that a correlation of test scores across the two measurement time points was not only due to consistency in the rank order of participants (ICC 3,1) but to agreement in absolute scores (ICC 2,1). Classic conventions for the evaluation of reliability estimates classify coefficients between .6 and .7 as moderate and borderline to insufficient (Evers, 2001). The test–retest correlations being lower than the estimates of internal consistency is in line with reports for other experimental measures of interpretation bias (Prieto-Fidalgo et al., 2022) and may be due to the measured biases varying over time. Current theoretical models of cognitive biases and their role in mental health emphasize that biases must not be thought of as stable traits but modes of information processing that can be (and should be) adapted to changing contextual demands (McNally, 2019; Parsons et al., 2016). This conceptualization of cognitive biases as context-dependent are in line with studies showing the ACT interpretation bias to be subject to situational context (Iigaya et al., 2016) and mood inductions (Lin et al., 2019).

Ultimately, the evaluation of measurement reliability should not solely be based on conventions. More important are the implications of measurement reliability for the statistical power of the study using the measures in question. Regarding correlation studies, it must be considered that the correlation between two variables cannot be higher than the root of the product of the reliability coefficients of the two variables. Thus, lower reliability in the measurement of one variable attenuates the correlations that can be observed between that variable and others. Let us assume, for example, that there is a true correlation of .3 between two variables A and B. Let us further assume that we can measure variable A with a reliability of .9. If we can also measure variable B with a reliability of .9, we expect to observe a correlation of .27. However, if we can only measure B with a reliability of .6, we expect to observe a correlation of .22. In the latter case, due to the less reliable measurement of one of the two variables, we would need a larger sample to detect the correlation at the same statistical threshold with the same statistical power. Otherwise, using the same sample size and statistical threshold, we have less statistical power to detect the existing association with our less reliable measurement.

Overall, we found an excellent internal consistency and an acceptable test–retest correlation for our experimental measure of inter-individual differences in interpretation bias. It must be emphasized that reliability is not a fixed quality of a particular task or paradigm. It is the property of a specific measurement using a task in a specific context on a specific sample of participants. In this sense, the estimates of reliability we calculated for our measurement with the ACT are not directly transferable to other contexts and samples. Nevertheless, we can expect that comparable applications of the ACT will result in similar reliability of measurement. While our findings do not release ourselves and other researchers from the need to calculate and report estimates of measurement reliability for each individual study, our study provides an important basis for selecting the ACT for future research. Our estimates of reliability can be used for power calculations and sample size planning. The high number of experimental trials we implemented in the ACT for the present study allows us to draw conclusions about the relation between ACT trial numbers and the resulting measurement reliability for the bias score. These analyses are intended to provide other researchers with a rough guide as to how many trials to include to achieve a certain level of measurement reliability.

## In a non-clinical sample, interpretation bias was not associated with trait markers of mental health

Previous studies, investigating interpretation biases with versions of the ACT in much smaller human samples, have occasionally reported associations with personality and trait markers of mental health, including reflective pondering, a type of rumination (Schick et al., 2013) and trait anxiety (Anderson et al., 2012; Paul et al., 2011; Schick et al., 2015). In our higher-powered study, however, we could not replicate these associations of interpretation bias with measures of depressiveness or anxiety. Across the ten self-report scales we included in our study, covering traits positively associated with mental health (*Mental Health +*: optimism, resilience, well-being, trait positive affect, behavioral activation) and negatively associated with mental health (*Mental Health -*: pessimism, depressiveness, anxiety, trait negative affect, behavioral inhibition) most correlations we observed were close to zero, not exceeding an effect size of *r* = |.11|, and statistically not significant when controlling for multiple comparisons. As – with sample sizes of only 20 to 67 participants – the previous studies reporting associations between an ACT interpretation bias and questionnaire measures were seriously underpowered, it has to be taken into consideration that their findings may represent false positives (Forstmeier et al., 2017; Ioannidis, 2005). In general, the demonstration of an association may be hindered by the fact that in the current study design the interpretation bias and the trait markers of mental health are assessed in different modalities: we are correlating an *implicit* measure of behavior shown in an experiment with *explicit* self-report from questionnaires. For this constellation, we know that correlations are typically low (Mischel, 1968; Bernoster et al., 2019).

From our results, we conclude that in non-clinical samples there are no or only weak associations to be expected between the ACT interpretation bias and trait markers of mental health. For some mental health-related traits, we observed small effects for an association with the ACT interpretation bias, which were in the expected direction but statistically not significant (e.g., *r* = – 10 for behavioral inhibition, *r* = – .11 for trait pessimism, *r* = .08 for trait resilience, and *r* =.11 for well-being). We cannot rule out that these effects are so small (and smaller than our pre-defined smallest effect size of interest of *r* = .15 for the current study) that they can only be reliably detected in even larger samples. Recent publications have emphasized that small effects are not necessarily meaningless. Consequences of small associations may cumulate across time and situations, possibly resulting in bigger effects, that practically matter, in the long run (Funder & Ozer, 2019; Götz et al., 2022). In this sense, even very small correlations between interpretation biases and mental health could lead to an accumulation of effects over time, with small differences in bias ultimately making a big difference for mental health. However, based on the current study, it is impossible to judge whether such a cumulation of consequences could exist for interpretation biases and mental health. It could just as well be the case that there are no accumulation effects or even counteracting mechanisms at work. Here, theoretical work is necessary that we see beyond the scope this paper. Models would need to be developed that explicitly state potentially amplifying or counteracting mechanisms so that suitable studies can be designed to test them (Anvari et al., 2023). In principle, small effects can especially be relevant when it comes to very important outcomes, among which we would include mental health in view of the associated personal and societal burdens. Mental health is a complex phenomenon, most likely determined by multiple causes, of which many may have only small effects (Götz et al., 2022). From a public health perspective, it may also be of value to better understand even the smaller causes if this results in approaches for interventions that can have a positive impact on mental health – with or without cumulation over time or people.

There may also be a range of variation in interpretation bias in non-clinical populations that is not associated with mental health, while only extreme levels of interpretation bias are. This hypothesis was examined in more detail comparing extreme groups of participants with particularly positive vs. particularly negative interpretation biases. Also here, we observed weak effects in the expected direction, that were, however, not significant when controlling for multiple comparisons (e.g., *d* = – .49 for depressiveness and *d* = .48 for well-being). As discussed above in the context of test–retest reliability, current theoretical considerations conceptualize cognitive biases as dynamic processes that are adapted to varying contexts. It is assumed that flexibility in cognitive biases is adaptive and as such normative in a non-clinical sample. Mood inductions and systematic variations in the situational context have been shown to affect interpretation biases in non-clinical samples (Iigaya et al., 2016; Lin et al., 2019). In the current study however, participants were not subjected to any mood induction. Under these conditions it may be less likely to find extreme biases beyond variation in the normative range. However, in psychopathology, cognitive biases might be more extreme and less adaptable, which could account for previous findings of difference in interpretation biases between patients with major depression and healthy participants (Everaert et al., 2017; Wessa, Armbruster-Genç et al., 2023).

## Constraints on generality

One limitation of the current study is the limited diversity of our convenience sample which was drawn from a “WEIRD”, i.e., mainly white, educated, industrialized, rich, and democratic population (Henrich et al., 2010). However, since the ACT uses abstract stimuli (geometric figures) that only gain affective meaning during the experimental procedure, it can be assumed that the perception and processing of the task is not or little dependent on socio-cultural learning histories. A study using the ACT in a sample of Chinese college students (Lin et al., 2019) showed similar effects of stimulus material and mood induction on interpretation bias as previous studies in European samples (Iigaya et al., 2016). This can be regarded as a positive indication that findings obtained using the ACT can be generalized to diverse populations. To what extent this also applies to the relations between interpretation bias, pessimism, depressiveness, and well-being that we observed in the present study needs to be investigated in future studies.

## Future perspectives

While our study shows that the ACT allows for a very reliable assessment of interpretation biases, its application in the laboratory is relatively time-consuming and dependent on the presence of the test person. To make the ACT easier to use for a routine diagnostic of interpretation bias, future research should investigate three potential adaptations of the task: (a) *Reduction of task application time*: Here, it should be tested to what extent providing explicit instructions on stimulus contingencies can reduce the acquisition time necessary for the ACT. (b) *Web-based implementation of the ACT*: An online implementation would allow for a highly standardized and time- as well as cost-efficient application of the ACT. This would also facilitate clinical assessment of interpretation bias to be used in personalized therapy for emotional disorders, esp. major depression. (c) *Omission of monetary reinforcement*: To make the applicability of the ACT independent of financial resources, it should be examined whether the task can also be used with virtual reinforcers, such as points and sounds, like they are used in computer games. These adaptions would also allow for an integration of the ACT into larger batteries of (neuro-)cognitive tests as an efficient measure of interpretation bias. Finally, to make the task applicable for individual diagnostic in clinical contexts, future research needs to establish normative data and define clinical cut-off values for determining clinically relevant expressions of interpretation bias.

Finally, previous research suggests that interpretation biases are affected by current mood and situational context (Iigaya et al., 2016; Lin et al., 2019). Theoretical models on the role of cognitive biases in resilience assume that the individual ability to flexibly adapt cognitive biases to varying contextual demands is indeed important for keeping up mental health (McNally, 2019; Parsons et al., 2016). Future studies should investigate the temporal dynamics of interpretation biases and their associations with well-being and psychopathology. The ACT is well suited for longitudinal studies because after an initial completion of the acquisition phase, the test phase can easily be repeated.

## Conclusion

Many experimental paradigms used to assess individual differences in cognitive biases have been criticized for providing low measurement reliability (Hedge et al., 2018; LeBel & Paunonen, 2011) – leading to problems of reproducibility in research on the role of cognitive biases in mental health (Parsons et al., 2019b). Here, we show that the Ambiguous Cue Task (ACT) allows for a highly reliable measurement of individual differences in approach-avoidance decisions for ambiguous affective information. In the non-clinical sample studied for the current investigation, associations between the ACT interpretation bias scores and mental health-related self-report measures of personality and well-being were generally small (*r* < |.15|) and statistically not significant. The task can be used to study interpretation biases in clinical samples (see, for example, Wessa, Armbruster-Genç et al., 2023) as well as in even bigger non-clinical samples, for which the small effect sizes of potential associations with measures of mental health must be taken into account (Götz et al., 2022). The ACT offers the possibility to calculate two different scores of interpretation bias – the ACT-IB-core and the ACT-IB-extended – which both showed excellent measurement reliability and a similar pattern of associations to mental health-related self-report measures. As the ACT-IB-extended considers more trials of the ACT (partly ambiguous in addition to fully ambiguous) it is more reliable for particularly short versions of the ACT that may be preferred in contexts, in which a most time efficient assessment is important (e.g., a 5-min version). As an *indirect* measure of interpretation biases the ACT is less susceptible to strategic responses of participants or effects of social desirability. As an *online* measure it records interpretations right at the time a person encounters ambiguous information, making it robust against memory effects or self-contingency bias often observed for retrospective or hypothetical reports on behavior in response to ambiguity. The ACT particularly stands out from other paradigms measuring interpretation bias in that it is suitable for translational research, allowing to study different levels of information processing involved in interpretation biases across species (e.g., cognitive, neurobiological, cellular; cf. Armbruster et al., 2012; Richter et al., 2014). The fact that the task uses abstract stimulus material that is associated with affective value only by a conditioning procedure within the task, ensures that the individual bias measures with the ACT is independent of pre-existing differences in affective value associated with more naturalistic stimuli like ambiguous facial expressions or social scenes. As such, the task is also well applicable across different socio-cultural contexts. In sum, with these features, the task represents a reliable tool for the assessment of interpretation biases and the investigation of their role as risk or resilience factors for emotional disorders in both basic as well as clinical research.

### Supplementary information

Below is the link to the electronic supplementary material.

Supplementary file1 (DOCX 442 KB)

## Data Availability

Data, task code and analyses code are available on OSF: https://osf.io/h5p89/?view_only=16d56cdd59424f0ea429bbb252801303
