# Supplementary material for: The Ambiguous Cue Task: Measurement reliability of an experimental paradigm for the assessment of interpretation bias and associations with mental health
Source: Behav Res Methods. 2024 Jul 12;56(7):7774–89. doi: 10.3758/s13428-024-02451-y (PMC11362423; doi:10.3758/s13428-024-02451-y)
Supplement: Supplementary file 1 — Supplementary file1 (DOCX 442 KB) [file 13428_2024_2451_MOESM1_ESM.docx]

**Supplemental Information**

**The Ambiguous Cue Task
- Measurement Reliability of an Experimental Paradigm for the Assessment of Interpretation Bias and Associations with Mental Health**

Diana J. N. Armbruster-Genç, Rebecca A. Rammensee, Stefanie M. Jungmann, Philine Drake, Michèle Wessa, Ulrike Basten

# Methods

Task instructions before the start of the **acquisition phase** in German (as presented in the study):

>>>

**Übungsphase**

Auf dem Bildschirm werden Ihnen **weiße Balken** gezeigt.
Sie sollen diese Balken als **Angebote** verstehen. Manche der Balken sind mit der Chance verbunden, **Geld zu gewinnen**, manche sind mit dem Risiko verbunden, **Geld zu verlieren**.
Die **Länge der Balken** signalisiert Ihnen, welche Balken "gut" und welche "schlecht" sind.
Pro Balken, der Ihnen präsentiert wird, geht es um 50 Cent.

**Mit der rechten Taste nehmen Sie einen Balken** (bzw. das damit verbundene Angebot) **an**, **mit der linken Taste lehnen Sie den Balken ab**.

Unmittelbar danach erhalten Sie eine Rückmeldung zu Ihrer Entscheidung.

Es gibt eine zusätzliche Herausforderung: Sie müssen zügig antworten. Wenn Sie nicht schnell genug antworten, verpassen Sie die Chance, einen Gewinn einzustreichen oder einen Verlust abzuwehren.

Machen Sie sich keine Gedanken, wenn Sie zu Beginn der Übungsphase zu langsam antworten oder ungünstige Entscheidungen treffen. Am Ende werden Sie mit der Aufgabe gut vertraut sein.

Die Übungsphase ist in Blöcke untergliedert, in denen Ihnen jeweils 30 Balken gezeigt werden. Sie werden mindestens 2 Blöcke absolvieren und so lange trainieren, bis Sie zu mindestens 90% günstige Entscheidungen treffen, d. h. "gute" Balken annehmen und "schlechte" Balken ablehnen.

**Die Übungsphase "zählt" noch nicht für Ihre Vergütung!**Sie dient Ihnen dazu, herauszufinden, wie Sie Ihre Gewinne und Verluste steuern können. Ihre **Vergütung richtet sich ausschließlich** nach dem Gewinn, den Sie in der anschließenden **Hauptaufgabe** erzielen.

*Wenn Sie Fragen haben, sprechen Sie bitte die Versuchsleitung an.*

***WEITER*** *mit der linken oder rechten Taste.*

<<<

English translation:

>>>

**Practice phase**

On the screen you will see **white bars**.

These should be understood as **offers**. Some bars are associated with the chance of **winning money**, some are associated with the risk of **losing money**. The **length of the bars** indicates which bars are "good" and which are "bad". Each bar that is presented to you is worth 50 cents.

**Use the right button to accept a bar** (or better: the associated offer), **use the left button to reject a bar**.

Immediately afterwards, you will receive feedback on your decision.

There is an additional challenge: You have to answer quickly. If you do not press the button in time, you might either miss the chance to secure a gain or to avoid a loss.

Don't worry if you answer too slowly or make unfavorable decisions at the beginning of the practice phase. By the end, you will be well acquainted with the task.

The practice phase is divided into blocks, in each of which you will be shown 30 bars. You will complete at least 2 blocks and you will practice until you make at least 90% favorable decisions, i.e., accept "good" bars and reject "bad" bars.

**This practice phase does not yet count towards your compensation!**

The practice phase just helps you to find out how to control your gains and losses. Your **remuneration** is based solely **on your gains in the subsequent main task**.

*If you have any questions, please talk to the study staff.*

***CONTINUE*** *with the left or right button.*

<<<

These task instructions were presented alongside this illustration:

**Figure S1.**


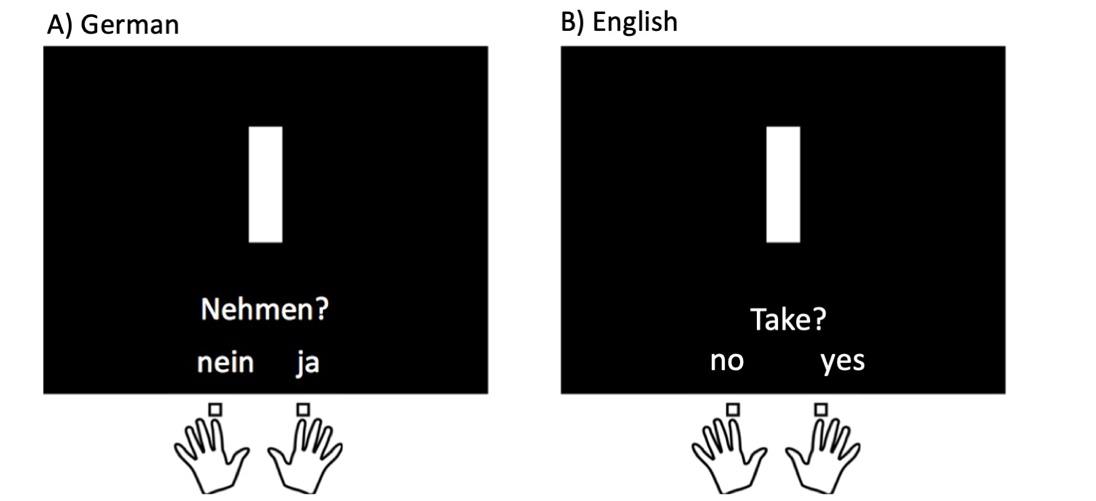


*Note.* A) Original screen display as used in the study, B) English translation.

Task instructions before the start of the **test phase** in German (as presented in the study):

>>>

**Hauptaufgabe**

Die Hauptaufgabe entspricht der Aufgabe in der Übungsphase. Es gibt lediglich zwei kleine Änderungen in der Bildschirmanzeige:

1) Die Tastenzuordnungen sind Ihnen mittlerweile bekannt. Daher werden sie nicht mehr unten auf dem Bildschirm angezeigt.
Eventuell erscheint Ihnen die Beurteilung der Balkenlängen dadurch etwas schwieriger.

2) Sie erhalten nun keine unmittelbare Rückmeldung mehr.

Die Hauptaufgabe dauert ca. 5 Minuten und ist in 4 Blöcke unterteilt.

**Die Hauptaufgabe "zählt" für Ihre Vergütung!**Es läuft ein Zähler im Hintergrund, der Ihre Gewinne und Verluste aufaddiert, so dass Ihnen am Ende Ihr Gesamtergebnis angezeigt und ausgezahlt werden kann. Sie können in dieser Aufgabe **bis zu 12,- Euro gewinnen!**

**2 Durchgänge**Sie werden die **Hauptaufhabe zweimal bearbeiten**. Am Ende zahlen wir Ihnen den Gewinn für den Durchgang aus, in dem Sie den höheren Gewinn erzielt haben.

*Wenn Sie Fragen haben, sprechen Sie bitte die Versuchsleitung an.*

***WEITER*** *mit der linken oder rechten Taste.*

<<<

English translation:

>>>

**Main task**

The main task corresponds to the task in the practice phase. There are only two small changes in the screen display:

1) You are now familiar with the key assignments. Therefore, they are no longer displayed at the bottom of the screen.
This may make it a little more difficult for you to assess the bar lengths.

2) You will no longer receive immediate feedback.

The main task takes approx. 5 minutes and is divided into 4 blocks.

**The main task "counts" towards your remuneration!**

A counter runs in the background, which adds up your gains and losses so that your overall result can be displayed and paid out to you at the end. You can win **up to 12 Euros in this task!**

**2 runs**You will complete the main task twice. At the end, we will pay you the gains for the round in which you have achieved the higher winnings.

*If you have any questions, please talk to the study staff.*

***CONTINUE*** *with the left or right button.*

<<<

These task instructions were presented alongside this illustration:

**Figure S2.**

**
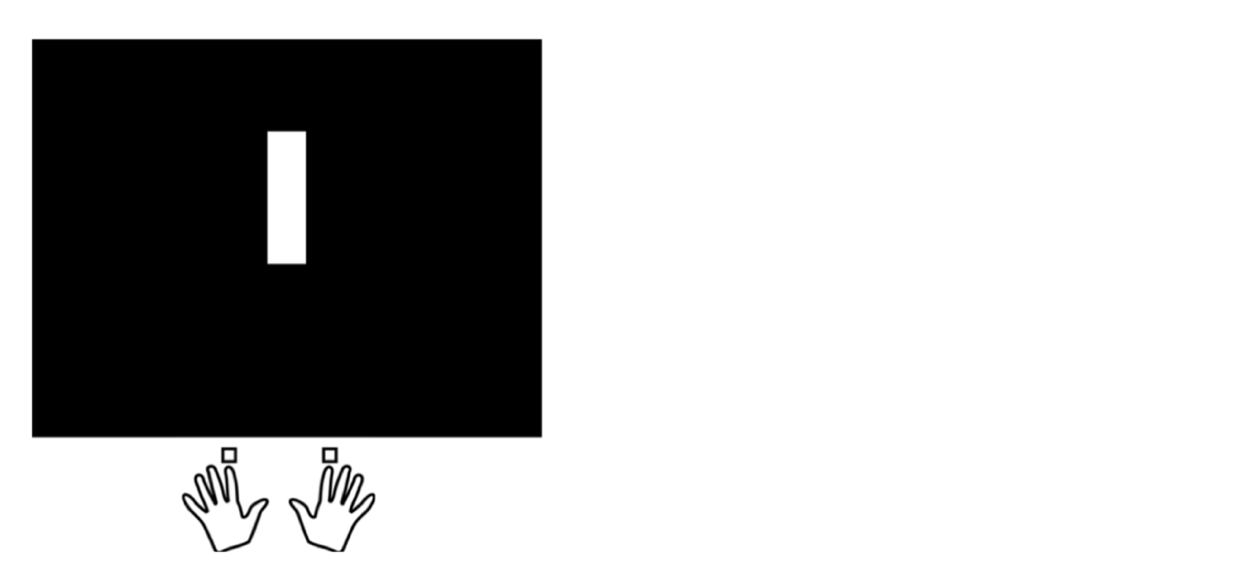
**

# Results

**Table S1** *Split-half Correlation Coefficients Depending on Total Trial Number.*

|  | **ACT-IB-core** | **ACT-IB-extended** |
| --- | --- | --- |
| **Trial number** | ***r*_SB_** | ***r*_SB_** |
| **60** | .69 | .82 |
| **100** | .79 | .89 |
| **140** | .85 | .92 |
| **180** | .88 | .94 |
| **220** | .91 | .96 |

**Figure S3***Test-Retest Scores from the Ambiguous Cue Task.*
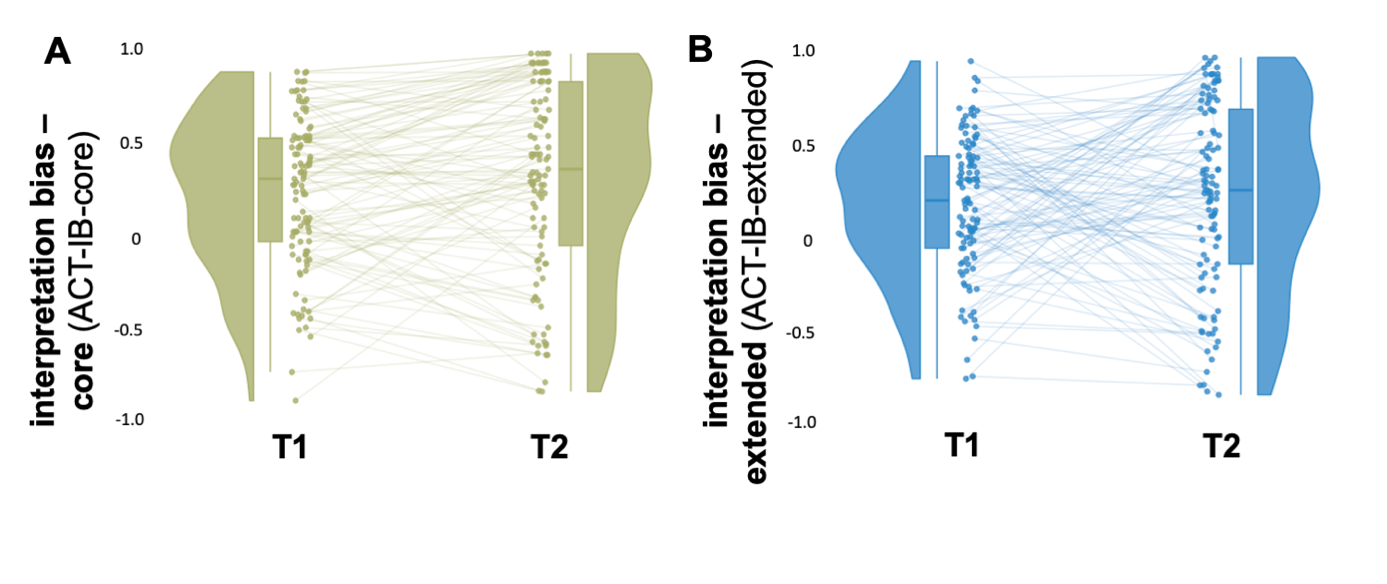
*Note*. Test (T1) and retest (T2) scores for **A**: Interpretation bias - core (ACT-IB-core) based on only fully ambiguous stimuli (AM) and **B**: Interpretation bias – extended (ACT-IB-extended) based on fully and partly ambiguous stimuli (AM, NP, NN).

**Table S2***Correlations Between Mental Health-Related Questionnaire Measures and ACT Interpretation Bias* *Scores*

|  | **Interpretation Bias – *core***  **(ACT)**  fully ambiguous trials | | |  | **Interpretation Bias – *extended***  **(ACT)**  fully and partly ambiguous trials | | |
| --- | --- | --- | --- | --- | --- | --- | --- |
|  | *r* | *p* | 95% CI |  | *r* | *p* | 95% CI |
| **Mental Health + Measures** |  |  |  |  |  |  |  |
| Trait Optimism (LOT-R) | .03 | .579 | [-.08;.13] |  | .03 | .636 | [-.08; .13] |
| Trait Resilience (RS-25) | .07 | .206 | [-.04; .17] |  | .08 | .159 | [-.03; .18] |
| Well-Being (WHO-5) | .09 | .110 | [-.02; .19] |  | .11 | .048 | [.0007; .21] |
| Trait Positive Affect (PANAS) | -.04 | .410 | [-.15; .06] |  | -.04 | .436 | [-.15; .06] |
| Behavioural Activation (BAS) | -.07 | .173 | [-.18; .03] |  | -.07 | .218 | [-.17; .04] |
| **Mental Health – Measures** |  |  |  |  |  |  |  |
| Trait Pessimism (LOT-R) | -.11 | .045 | [-.21; -.002] |  | -.09 | .083 | [-.20; .01] |
| Depressiveness (STADI) | -.04 | .446 | [-.14; .06] |  | -.04 | .466 | [-.14; .07] |
| Anxiety (STADI) | -.01 | .833 | [-.12; .09] |  | -.01 | .850 | [-.11; .09] |
| Trait Negative Affect (PANAS) | -.06 | .300 | [-.16; .05] |  | -.04 | .480 | [-.14; .07] |
| Behavioural Inhibition (BIS) | -.09 | .096 | [-.19; .02] |  | -.10 | .060 | [-.20; .004] |

*Note.* Pearson correlation coefficients, *p*-values, and 95% confidence intervals for correlations with interpretation bias *– core* (ACT-IB-core) and interpretation bias *– extended* (ACT-IB-extended) illustrated in Figure 4. Self-report measures are grouped into those that are conceptually linked to mental health in a positive (Mental Health +) vs. negative (Mental Health -) way.

**Table S3**

*Comparison of Mental Health-Related Self-Report Measures for Groups with Extreme Positive vs. Negative Interpretation Biases (ACT-IB-Extended)*

|  | **Extreme negative ACT-IB-ext**  ***n* = 36** | | **Extreme positive bias ACT-IB-ext**  ***n* = 35** | |  | **Group comparison** | | | |
| --- | --- | --- | --- | --- | --- | --- | --- | --- | --- |
|  | *Mean (SD)* | | *Mean (SD)* | |  | *t(df)* | *p* | 95% CI | *d* |
| **Mental Health +** |  |  | |  |  |  |  |  |  |
| Trait Optimism (LOT-R) | 8.26 (2.49) | | 8.22 (2.49) | |  | .06 (69) | .953 | [-1.14; 1.21] | -.01 |
| Trait Resilience (RS-25) | 126.26 (18.81) | | 132.86 (20.62) | |  | -1.41 (69) | .163 | [-15.95; 2.75] | .34 |
| Well-Being (WHO-5) | 12.60 (4.03) | | 14.56 (4.40) | |  | -1.95 (69) | .055 | [-3.96;.04] | .47 |
| Trait Positive Affect (PANAS) | 34.69 (6.67) | | 35.44 (6.22) | |  | -.50 (69) | .622 | [-3.81; 2.30] | .12 |
| Behavioural Activation (BAS) | 3.15 (.40) | | 3.09 (.35) | |  | .60 (69) | .553 | [-.12; .23] | -.14 |
| **Mental Health –** |  |  | |  |  |  |  |  |  |
| Trait Pessimism (LOT-R) | 4.31 (2.54) | | 4.03 (1.98) | |  | .53 (69) | .600 | [-.79; 1.36] | -.13 |
| Depressiveness (STADI) | 19.51 (4.66) | | 18.17 (4.69) | |  | 1.22 (69) | .223 | [-.86; 3.56] | -.29 |
| Anxiety (STADI) | 21.37 (6.61) | | 21.03 (5.88) | |  | .23 (69) | .817 | [-2.61; 3.30] | -.06 |
| Trait Negative Affect (PANAS) | 18.40 (6.35) | | 17.72 (7.00) | |  | .43 (69) | .671 | [-2.49; 3.84] | -.10 |
| Behavioural Inhibition (BIS) | 2.89 (.38) | | 2.78 (.42) | |  | 1.14 (69) | .259 | [-.08; .30] | -.27 |

Groups did not differ significantly in age (*t*(67) = 1.05, *p* = .298) or sex (*Χ*^2^(1) = 0, *p* = 1.00).
